# Risk factors for hematuria during indwelling urinary catheterization in acute myocardial infarction: a comparative analysis using logistic regression and decision tree

**DOI:** 10.3389/fcvm.2025.1597796

**Published:** 2025-11-14

**Authors:** Jia Zhang, Song Bin Huang, Dan Ni Peng, Jin Huang, Yan Jie Chen, Ling Ling Yang

**Affiliations:** Heyuan People’s Hospital, Heyuan, China

**Keywords:** acute myocardial infarction, hematuria, logistic regression, decision tree, machine learning

## Abstract

**Objective:**

This study aimed to systematically identify risk factors for urinary catheter-related hematuria in patients with acute myocardial infarction (AMI). By integrating logistic regression and decision tree models, we sought to develop actionable strategies for risk stratification and complication prevention.

**Methods:**

A retrospective analysis of 209 AMI patients was conducted to evaluate predictors of hematuria, including demographics, coagulation indices (INR, platelets), and procedural variables. Logistic regression and decision tree (CART algorithm) models were employed to identify risk factors and their interactions. Model performance was assessed using ROC-AUC, sensitivity, and specificity.

**Results:**

The incidence of catheter-related hematuria was 32.5%. Both models identified persistent agitation during catheter indwelling and PLT ≤ 246 as common predictors. The logistic regression model specifically identified Gender (OR = 0.202), patient awareness of catheter purpose and precautions (OR = 0.470), and emergency catheter placement (OR = 2.257) as significant factors. The decision tree model uniquely identified INR > 0.955 and repeated complaints of urethral pain as predictors.

**Conclusion:**

Hematuria in AMI patients results from coagulation dysfunction, procedural trauma, and behavioral factors. The combined use of logistic regression and decision trees enhances risk stratification. Clinical strategies should prioritize gentle catheterization, dynamic coagulation monitoring, and patient education to reduce complications.

## 1 Background

According to the Global Burden of Disease Study 2021, acute myocardial infarction (AMI) is the leading cause of death worldwide, with approximately 8.6 million deaths per year, accounting for 44.7% of total deaths from cardiovascular diseases (1).While contemporary guidelines emphasize rapid re-vascularization and anti-thrombotic therapy (2, 3), yet critical iatrogenic risks like catheter-related hematuria remain unaddressed in these guidelines.

At present, nursing studies for AMI patients mostly focus on the construction and optimization of early rehabilitation interventions (4), psychosocial support (5), and standardized complication prevention and control pathways (6). However, despite the important clinical value of indwelling urinary catheterization in the management of critically ill patients, there are still significant gaps in systematic studies on complications associated with indwelling urinary catheterization in AMI patients. Existing literature shows that complications such as hematuria and urinary tract infection may occur during indwelling urinary catheterization, but relevant studies are mostly limited to common inpatients (7, 8). It is worth noting that the risk of catheterism-related hematuria in AMI patients may be significantly higher than that of the general population due to special pathophysiological states such as high-intensity anticoagulation and antiplatelet therapy and hemodynamic instability (9). Hematuria not only exacerbates patient distress but may necessitate anticoagulant cessation, jeopardizing reperfusion efficacy (10). Notably, the 2023 ESC (2) and 2025 ACC/AHA (3) lack recommendations on catheter-complication prevention.

Conventional statistical approaches (e.g., logistic regression) often fail to capture clinically critical interactions, particularly in AMI patients where anticoagulant-ischemia-procedure interactions dominate hematuria pathogenesis. Decision tree models address this by identifying threshold-based risk strata (11), but their utility in AMI-specific iatrogenic injury prediction remains untested.

Therefore, this study pursues dual objectives: (1) Primarily, to identify clinically modifiable risk factors for catheter-related hematuria in AMI patients through multidimensional analysis of demographic, procedural, and laboratory variables. (2) Secondarily, to compare the predictive performance and complementary value of logistic regression vs. decision tree models for clinical risk stratification.

We hypothesize that machine learning approaches more effectively characterize nonlinear risk interactions specific to AMI pathophysiology.

## 2 Methods

### 2.1 Study design and participants

A convenience sample of 209 AMI patients admitted to a tertiary hospital cardiovascular center (December 2020–December 2022) was retrospectively analyzed. Inclusion criteria: (1) AMI diagnosis per Chinese guidelines; (2) ST-elevation myocardial infarction; (3) indwelling catheterization during hospitalization. Exclusion criteria: (1) preexisting hepatic/hematologic disorders, malignancy, or non-AMI anticoagulant use; (2) coma. This study was approved by the Institutional Review Board of Heyuan People's Hospital (Approval No. 2021-RE-026). Written informed consent was obtained from all individual participants or their legal guardians prior to data collection.

### 2.2 Variables and measurements

The binary outcome was hematuria occurrence (yes/no). Hematuria was objectively defined as the presence of ≥5 red blood cells per high-power field (RBCs/HPF) on microscopic examination of a centrifuged urine specimen (12). Any documented episode of gross hematuria in the medical record was also considered a positive outcome. Twenty-four covariates spanning demographics, urological history, catheterization procedural details, and laboratory indices (e.g., PLT, INR, PT) were evaluated. To ensure methodological rigor, we operationalized key variables through standardized definitions detailed in Table 1. This standardization enabled consistent data collection across clinical scenarios. According to the method of sample size calculation, the sample size was obtained from 5 to 10 times of the number of variables (15), and the sample size was calculated to be 120–240 cases.

**Table 1:** Operational definitions of key variables.

Variable	Definition	Assessment method
Emergency catheter placement	Order-to-insertion time ≤10 min	Electronic Medical Record timestamp audit
Acute agitation during catheter insertion	RASS≥+2 (13) OR volitional catheter contact (from urethral meatus contact to balloon inflation)	Real-time nurse observation
Indwelling catheterization process smoothly	Catheter placement achieving all of the following: (1) first-attempt success (2) no procedural interruption >30 s (3) nurse-rated smoothness of “smooth” or “very smooth”	Nurse documentation and post-procedure questionnaire
Persistent agitation during catheter indwelling	≥2 episodes of RASS ≥+2 in 24 h OR volitional catheter contact (post-inflation to removal)	Real-time nurse observation
Urinary fixed properly	Zero hazardous tension: (1) No visible urethral meatus traction; (2) Drainage bag weight fully supported (not dependent on tubing).	Bedside nurses assessment per 8 h
Repeated complaints of urethral pain	Frequency & Severity: (1) ≥3 discrete episodes in 24 h; (2) Each episode: Numeric Rating Scale (NRS) ≥4 (14)	NRS screening per 8 h
Patient was aware of the purpose and precautions of the indwelling catheter	The patient demonstrated two components: (1) Correct language expression of preventive measures; (2) Display of protective measures and hazard identification	Knowledge assessment and behavioral observation

### 2.3 Bias mitigation

To address limitations of the retrospective design, we implemented a tripartite bias mitigation protocol: (1) Procedural standardization was enforced using the CAUTI bundle guidelines (16), ensuring consistent catheter insertion/maintenance techniques across all cases; (2) Blinding of outcome assessors to predictor variable status prevented detection bias during hematuria adjudication; (3) Multivariable adjustment for clinically relevant confounders (age and renal function) minimized residual confounding in regression models.

### 2.4 Statistical analysis

Complete variable assignment details (including coding logic for all 24 covariates) are documented in Supplementary Table S1. Continuous variables were compared via *t*-tests; categorical variables via *χ*^2^ or Fisher's exact tests.

All candidate variables with a univariate *P* < 0.1 were entered into the logistic regression and decision tree model. Multicollinearity was assessed using the variance inflation factor (VIF), with a threshold of VIF >5 indicating severe collinearity. Due to severe collinearity between INR and PT (VIF >10), one of them had to be excluded from the multivariate logistic regression model to ensure stability and interpretability. We opted to retain PT and exclude INR for the following reasons: (1) PT, as a direct measure of clotting time in seconds, offers more intuitive clinical interpretability in our STEMI cohort managed primarily with antiplatelet therapy; (2) PT represents the fundamental measurement from which INR is derived, making it a more robust and statistically stable choice for regression analysis. It is important to note that the clinically relevant information contained in INR was subsequently captured by the decision tree model, which identified a threshold of INR >0.955, thus demonstrating the complementary nature of our two-model approach.

Decision tree modeling employed the Classification and Regression Trees (CART) algorithm with Gini impurity minimization as the splitting criterion. Key parameters included: (1) Maximum tree depth: 5; (2) Minimum cases in parent node: 50; (3) Minimum cases in child node: 5; (4) Pruning via cost-complexity pruning (*α* = 0.01) and 10-fold cross-validation. Model performance was assessed via ROC-AUC, sensitivity, specificity, and Youden's index.

## 3 Result

### 3.1 Basic characteristics

Among 209 AMI patients with indwelling urinary catheters, hematuria occurred in 68 cases (32.5%). As detailed in Table 2, significant risk factors encompassed behavioral aspects such as persistent agitation during catheterization (57.97% vs. 20.00%, *P* < 0.001), emergency catheter placement (45.24% vs. 24.00%, *P* = 0.001), and repeated urethral pain complaints (52.94% vs. 22.70%, *P* < 0.001), demographic differences with higher incidence in males (39.13% vs. 19.72% in females, *P* = 0.005), as well as coagulation indicator characterized by elevated platelets (*P* = 0.003) and lower INR (*P* = 0.094). No significant associations were observed for age, cystitis history, catheter material, or thrombolytic therapy (*P* > 0.1) (17).

**Table 2:** General and hematuria-related characteristics of the study participants (*N* = 209).

Categories	Total	No hematuria (*n* = 141)	Hematuria (*n* = 68)	*χ*^2^/*t*	*P*
	*n* (%) or Mean ± SD	*n* (%) or Mean ± SD	*n* (%) or Mean ± SD		
Age				2.131	0.345
<60	49 (23.44)	33 (67.35)	16 (32.65)		
60–75	81 (38.76)	59 (72.84)	22 (27.16)		
>75	79 (37.80)	49 (62.03)	30 (37.97)		
Gender				8.048	**0** **.** **005**
Male	138 (66.03)	84 (60.87)	54 (39.13)		
Female	71 (33.97)	57 (80.28)	14 (19.72)		
Cystitis				0.300	0.584
No	158 (75.6)	105 (66.46)	53 (33.54)		
Yes	51 (24.4)	36 (70.59)	15 (29.41)		
History of hematuria (last six months)				0.493	0.483
No	147 (70.33)	97 (65.99)	50 (34.01)		
Yes	62 (29.67)	44 (70.97)	18 (29.03)		
History of urethral surgery				0.489	0.484
No	148 (70.81)	102 (68.92)	46 (31.08)		
Yes	61 (29.19)	39 (63.93)	22 (36.07)		
Urinary tract infections				0.002	0.966
No	151 (72.25)	102 (67.55)	49 (32.45)		
Yes	58 (27.75)	39 (67.24)	19 (32.76)		
Urinary systemmatic calculi				1.238	0.266
No	127 (60.77)	82 (64.57)	45 (35.43)		
Yes	82 (39.23)	59 (71.95)	23 (28.05)		
Urinary tube specification					1
16Fr	203 (97.13)	137 (67.49)	66 (32.51)		
<16Fr	6 (2.87)	4 (66.67)	2 (33.33)		
Emergency catheter placement				10.324	**0** **.** **001**
No	125 (59.81)	95 (76.00)	30 (24.00)		
Yes	84 (40.19)	46 (54.76)	38 (45.24)		
Catheter material				1.221	0.269
Silica gel	128 (61.24)	90 (70.31)	38 (29.69)		
Latex	81 (38.76)	51 (62.96)	30 (37.04)		
Acute agitation during catheter insertion				0.365	0.546
No	162 (77.51)	111 (68.52)	51 (31.48)		
Yes	47 (22.49)	30 (63.83)	17 (36.17)		
Indwelling catheterization process smoothly				1.301	0.254
No	101 (48.33)	72 (71.29)	29 (28.71)		
Yes	108 (51.67)	69 (63.89)	39 (36.11)		
Persistent agitation during catheter indwelling				30.36	**0** **.** **000**
No	140 (66.99)	112 (80.00)	28 (20.00)		
Yes	69 (33.01)	29 (42.03)	40 (57.97)		
Urinary fixed properly				1.229	0.268
No	119 (56.94)	84 (70.59)	35 (29.41)		
Yes	90 (43.06)	57 (63.33)	33 (36.67)		
Repeated complaints of urethral pain				19.120	**0** **.** **000**
No	141 (67.46)	109 (77.30)	32 (22.70)		
Yes	68 (32.54)	32 (47.06)	36 (52.94)		
Patient was aware of the purpose and precautions of the indwelling catheter				6.745	**0.009**
No	93 (44.50)	54 (58.06)	39 (41.94)		
Yes	116 (55.50)	87 (75.00)	29 (25.00)		
Thrombolytic therapy				0.005	0.944
No	139 (66.51)	94 (44.98)	45 (21.53)		
Yes	70 (33.49)	47 (22.49)	23 (11.00)		
Treatment				1.183	0.553
Primary PCI	121 (57.89)	78 (64.46)	43 (35.54)		
Post-thrombolysis PCI	70 (33.5)	50 (71.43)	20 (28.57)		
Post-thrombolysis CAG	18 (8.6)	13 (72.22)	5 (27.78)		
PLT (*10^9^)	236.172 ± 78.382	213.118 ± 63.941	247.291 ± 82.392	3.01	**0** **.** **003**
PT(s)	13.409 ± 1.968	13.989 ± 3.108	13.129 ± 0.942	−2.231	**0** **.** **029**
INR	1.061 ± 0.589	1.202 ± 1.014	0.993 ± 0.094	−1.699	**0** **.** **094**
APTT(s)	42.036 ± 34.376	45.535 ± 38.725	40.348 ± 32.081	−1.022	0.308
FIB	5.55 ± 19.961	4.09 ± 1.435	6.25 ± 24.279	0.731	0.466
TT	24.02 ± 32.267	27.60 ± 38.777	22.30 ± 28.601	−1.114	0.267

Percentages in No Hematuria and Hematuria columns represent proportions within each category.

Bold values indicate *P* < 0.1.

*P* < 0.05 was considered statistically significant.

CAG, coronary angiography; PCI, percutaneous coronary intervention bold; PLT, platelet count; PT, prothrombin time; INR, International Normalized Ratio; APTT, activated partial thromboplastin time; FIB, fibrinogen; TT, thrombin time.

### 3.2 Logistic regression analysis of factors affecting hematuria in patients with AMI during indwelling urinary catheterization

Using whether hematuria occurred as the dependent variable (coded as 0 = no, 1 = yes), variables with statistical significance in univariate analysis (*P* < 0.1) were included in the logistic regression model. The results of multivariate logistic regression analysis showed that emergency catheter placement, persistent agitation during catheter indwelling, and repeated complaints of urethral pain were risk factors for hematuria. Gender, patient awareness of the purpose and precautions of the indwelling catheter, PLT (*10^9^), and INR were protective factors for hematuria, as detailed in Table 3.

**Table 3:** Logistic regression analysis of hematuria during catheter indentation in patients with acute myocardial infarction.

Variate	B	S.E	Wald	OR (95% CI)	*P*
Gender	−1.597	0.456	12.245	0.202 (0.083–0.495)	0.000
Emergency catheter placement	0.814	0.373	4.757	2.257 (1.086–4.692)	0.029
Persistent agitation during catheter indwelling	1.617	0.411	15.461	5.039 (2.250–11.285)	0.000
Repeated complaints of urethral pain	1.15	0.405	8.067	3.158 (1.428–6.983)	0.005
Patient was aware of the purpose and precautions of the indwelling catheter	−0.755	0.378	3.996	0.470 (0.224–0.985)	0.046
PLT (×10^9^/L)	−0.008	0.003	7.181	0.992 (0.987–0.998)	0.007
PT(s)	0.407	0.142	8.153	1.502 (1.136–1.986)	0.004
Constant	−5.049	2.021	6.243	0.006	0.012

INR was excluded from the final model due to severe multicollinearity with PT (see Methods for details).

### 3.3 Performance metrics and decision tree architecture of prediction models

#### 3.3.1 Head-to-head performance metrics

**Figure 1:**
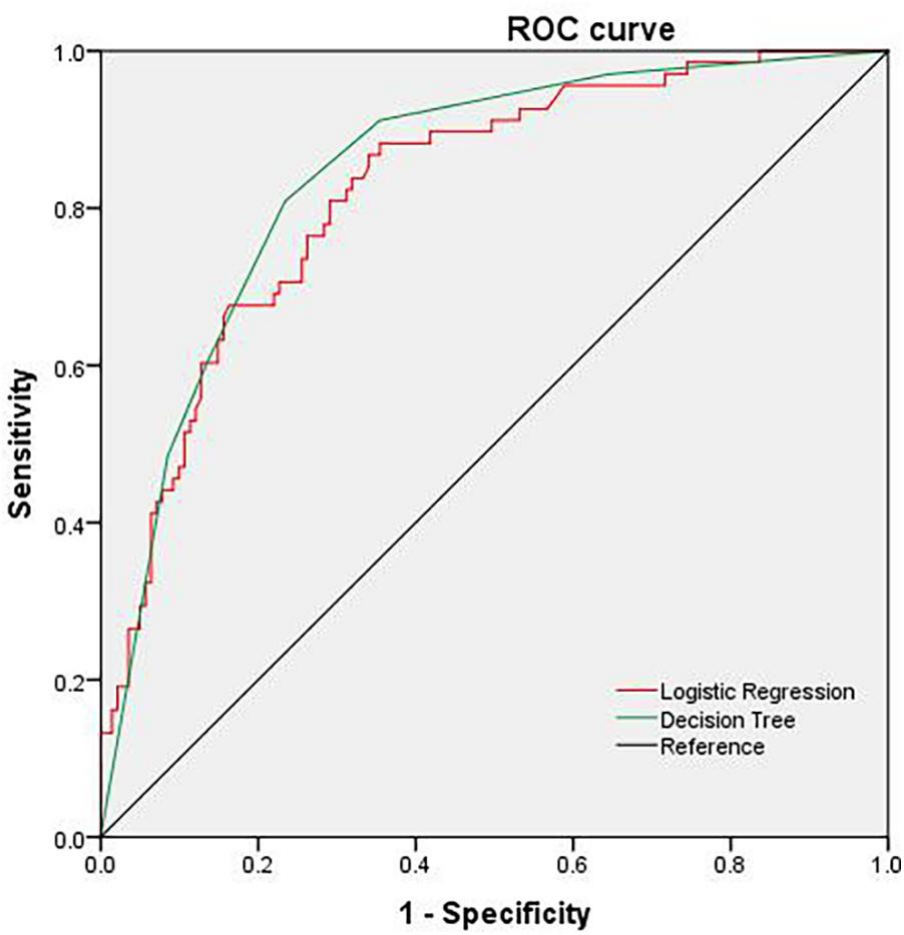
ROC curve predicted by decision tree and logistic regression model.

As detailed in Table 4 and Figure 1, both models demonstrated robust predictive capability for hematuria. The logistic regression model achieved higher sensitivity (86.8% vs. 80.9%), while the decision tree model offered superior specificity (76.6% vs. 66.0%). The decision tree also yielded a marginally higher area under the ROC curve (AUC = 0.845, 95% CI: 0.789–0.900) compared to the logistic regression model (AUC = 0.825, 95% CI: 0.766–0.883), a difference that was statistically significant.

**Table 4:** Comparison of analysis effect between logistic regression model and decision tree model.

Model	AUC	S.E.	95% CI	*P*
Logistic	0.825	0.03	0.766–0.883	0.000
Decision Tree	0.845	0.028	0.789–0.9	0.000

#### 3.3.2 Decision tree architecture and risk stratification

**Figure 2:**
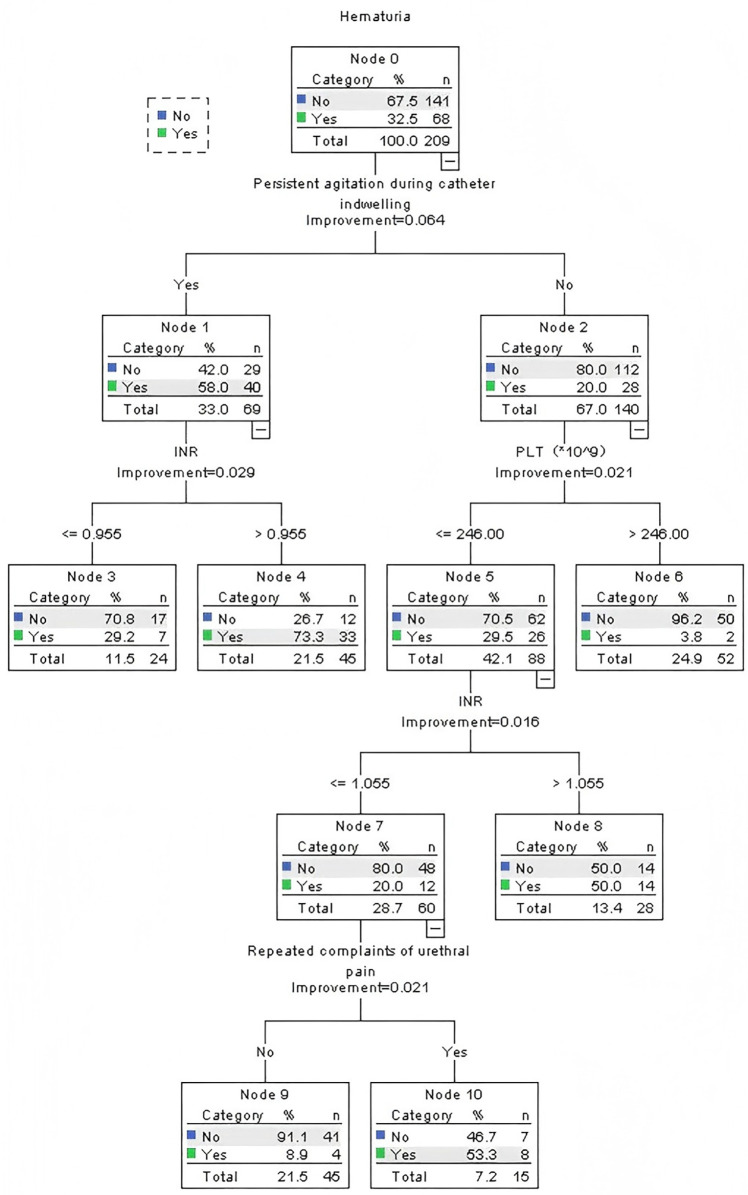
Classification and regression tree model (*N* = 209).

Beyond conventional metrics, the decision tree provided clinically actionable, granular risk stratification that logistic regression cannot directly offer. The CRT algorithm (Figure 2) identified persistent agitation during catheter indwelling as the primary split node (Gini importance = 41.2%), establishing it as the most influential predictor. The model generated three distinct risk stratification pathways: (1) High-risk pathway (*n* = 30, 14.4% of cohort): Patients with persistent agitation and INR >0.955 had a 73.3% probability of hematuria. (2) Intermediate-risk pathway (*n* = 28, 13.4% of cohort): Patients without agitation but with PLT ≤ 246 × 10^9^/L and INR >1.055 had a 50.0% probability of hematuria. (3) Low-risk pathway (*n* = 151, 72.2% of cohort): Patients without agitation, with PLT >246 × 10^9^/L, and no repeated urethral pain had a 3.8% probability of hematuria.

The relative importance of the predictors, as quantified by the normalized Gini decrease, was: persistent agitation (41.2%) > INR (28.5%) > platelet count (19.7%) > repeated urethral pain (10.6%).

## 4 Discussion

### 4.1 Key findings and mechanistic interpretations

The primary objective of this study was to identify clinically modifiable risk factors for catheter-related hematuria in AMI patients. Our analysis reveals a multifactorial etiology, wherein the interplay of coagulation dysfunction, procedural trauma, and behavioral factors creates a distinct high-risk phenotype. The incidence of catheter-related hematuria in this AMI cohort was 32.5%, which is strikingly elevated compared to the rate of 8%–15% reported in general inpatient populations (7, 18). This disparity can be attributed to the unique pathophysiological profile of AMI patients, which is characterized by a high-risk interplay of coagulation impairment, procedural vulnerability, and hemodynamic compromise. For instance, reduced cardiac output in AMI may lead to bladder microcirculation disorders, and resultant mucosal ischemia could potentially exacerbate mechanical injury from catheter friction (19). The modifiable risk factors identified in this study can be conceptually organized into three key mechanistic domains.

#### 4.1.1 Procedural and behavioral factors: the most potent predictors

1. Persistent Agitation: Logistic regression analysis showed that the odds ratio (OR) for persistent agitation during catheter indwelling was 5.586 (*P* < 0.001). The decision tree model further identified it as the primary split node (Gini index = 0.32), contributing 41% of the model's explanatory power, underscoring the critical role of minimizing mechanical trauma through adequate sedation or non-pharmacological calming techniques. The pathological mechanism lies in the fact that agitation caused by pain or anxiety can exacerbate mechanical friction between the catheter and the urethral mucosa, directly damaging vascular endothelial cells (7). For high-risk patients (e.g., those with an pain score >4), it is recommended to administer a low dose of dexmedetomidine 10 min before catheterization. Low-dose dexmedetomidine can significantly reduce the incidence of postoperative catheter-related bladder discomfort (CRBD). Randomized controlled trials have shown that dexmedetomidine reduces the incidence of Catheter-related Bladder Discomfort (CRBD) by 55% (20), visualized procedural education or adequate communication can reduce the risk of catheter-related complications (7).
2. Emergency Catheter Placement: Emergency procedures significantly increased the risk (OR = 2.257, *P* = 0.029). Emergency operations often lead to insufficient lubrication and over-rapid balloon inflation (21) due to time constraints, increasing the risk of mucosal tearing (16). The European Association of Urology (EAU 2023) states that catheters must be adequately lubricated to minimize urethral damage during emergency catheterization. Sterile water-based lubricants should be preferred (22).
3. Patient Awareness: Patient awareness of the purpose and precautions of the indwelling catheter was a significant protective factor, associated with a 53% reduction in the risk of hematuria (OR = 0.470, *P* = 0.046). This presents a low-cost, high-impact intervention strategy. While the decision tree does not reflect such social behavioral factors because it focuses more on the stratification of physiological indicators.

#### 4.1.2 Coagulation dysfunction: complementary insights from two modelling approaches

Our analysis robustly validates coagulation dysfunction as a central mechanism for hematuria, with logistic regression and decision tree models providing distinct yet complementary evidence.

The logistic regression model, isolating independent effects, identified Prolonged Prothrombin Time (PT) as a significant linear risk factor (OR = 1.502, *P* = 0.004), quantitatively confirming that anticoagulation therapy steadily increases hemorrhagic risk. The decision tree, designed to capture thresholds and interactions, pinpointed INR >0.955 as a critical classifier for high-risk patients, specifically when combined with patient agitation.

The use of PT in regression and INR in the tree is not a discrepancy but a reflection of their mechanistic redundancy and model purposes. PT provides the general, adjusted effect of coagulopathy, while INR provides a specific, clinically actionable threshold for rapid triage. Together, they offer a complete picture: coagulopathy is a fundamental driver of risk, and its danger is acutely amplified in agitated patients even at a marginally elevated INR.For high-risk patients with both elevated INR (>0.95) and decreased PLT (<150 × 10^9^/L), a combined intervention strategy is required: ① Dynamic Monitoring: Use thromboelastography (TEG) to assess overall coagulation function, replacing single INR or PLT measurements. Adjust the intensity of anticoagulation dynamically based on the R value of TEG (8–12 min) to reduce the risk of excessive inhibition of coagulation factors (23). ② Stratified Management: When PLT <150 × 10^9^/L, increase the prophylactic platelet transfusion threshold from 10 × 10^9^/L to 50 × 10^9^/L (24).

#### 4.1.3 Demographic and other factors

Gender paradox: This study found that the risk of catheter-related hematuria in male patients was significantly lower than that in females (OR = 0.202, *P* < 0.001), which contrasts with the conventional anatomical view that male catheterization carries higher inherent risk. While this observed association requires further confirmation, we hypothesize that it could be explained by a combination of behavioral and pathophysiological factors, rather than anatomy alone. Several non-mutually exclusive hypotheses could be proposed to explain this finding: (1) Potential Operator Bias: Informal interviews with nursing staff suggested a perception of higher risk during male catheterization, which might lead to more cautious techniques (25) (e.g., gentler insertion, more generous lubrication) that could mitigate trauma. However, this was not objectively measured in our study. (2) Potential Anatomical and Pharmacological Interactions: The shorter female urethra could potentially be more susceptible to irritation from catheter movement (26). Furthermore, existing literature indicates that female AMI patients on clopidogrel may have a higher incidence of hyperplatelet reactivity (HPR) and an increased risk of mild bleeding events compared to males (27). It is plausible that the confluence of a more sensitive urethral anatomy and a sex-specific pharmacological profile could contribute to the elevated risk observed in females. The hypotheses outlined above regarding operator behavior and sex-specific pharmacodynamics are speculative and require rigorous validation in future prospective studies.

### 4.2 Synergistic model integration for enhanced clinical stratification

The integrative use of logistic regression and decision tree models provides a comprehensive and clinically actionable framework for hematuria risk stratification in AMI patients. While both models demonstrated robust predictive performance, they offer distinct and complementary insights: logistic regression quantifies the independent net effect of key variables (e.g., the protective role of patient education), while the decision tree model reveals critical interaction effects and threshold-based clinical pathways, enabling rapid triage. To translate these insights into bedside practice, we propose a sequential clinical workflow:

1. The process begins with rapid triage using the decision tree model (Figure 2). Clinicians can initially assess for Persistent Agitation to immediately identify the highest-risk cohort. For non-agitated patients, subsequent evaluation of Platelet Count and INR further stratifies them into intermediate- or low-risk pathways. This entire process leverages readily available data to assign a risk level rapidly.
2. This initial triage then directly informs targeted, model-guided interventions. For patients in the high-risk pathway, the logistic regression model underscores the critical need to mitigate Emergency Catheter Placement (OR = 2.257) and aggressively manage Persistent Agitation (OR = 5.039). For those at intermediate risk, the strong protective effect of Patient Awareness (OR = 0.470) highlights patient education as a key modifiable intervention. Furthermore, a signal like Repeated Urethral Pain across both models should trigger enhanced monitoring for complications.

This synergistic approach—using the decision tree for efficient stratification and the regression model for depth of intervention—bridges the gap between prediction and prevention, offering a pragmatic blueprint for clinical implementation.

### 4.3 Limitations

This study has the following limitations: Firstly, the single-center, retrospective design may lead to selection bias. Its research results still need to be validated in prospective, multi-center cohorts to enhance its universality. Secondly, our analysis did not stratify the risk of hematuria by clinical Settings (such as ICU and general wards), which may affect the occurrence and detection of hematuria.

## 5 Conclusion

This study systematically identified the risk profile for catheter-related hematuria in AMI patients using both logistic regression and decision tree models. Logistic regression highlighted the impact of behavioral and demographic factors such as gender, emergency catheterization, and patient education, while the decision tree model revealed critical interactions between coagulation function and procedural behavior. The complementary strengths of these models provide a comprehensive framework for clinical risk assessment, bridging “independent risk weights” with “stratified clinical pathways”. We recommend their combined use to optimize risk stratification and guide personalized intervention strategies.

## Data Availability

The raw data supporting the conclusions of this article will be made available by the authors, without undue reservation.
